# Strain/species identification in metagenomes using genome-specific markers

**DOI:** 10.1093/nar/gku138

**Published:** 2014-02-12

**Authors:** Qichao Tu, Zhili He, Jizhong Zhou

**Affiliations:** ^1^Department of Microbiology and Plant Biology, Institute for Environmental Genomics, University of Oklahoma, Norman, OK 73072, USA, ^2^Earth Science Division, Lawrence Berkeley National Laboratory, Berkeley, CA 94720, USA and ^3^State Key Joint Laboratory of Environmental Simulation and Pollution Control, School of Environment, Tsinghua University, Beijing 100084, China

## Abstract

Shotgun metagenome sequencing has become a fast, cheap and high-throughput technology for characterizing microbial communities in complex environments and human body sites. However, accurate identification of microorganisms at the strain/species level remains extremely challenging. We present a novel *k*-mer-based approach, termed GSMer, that identifies genome-specific markers (GSMs) from currently sequenced microbial genomes, which were then used for strain/species-level identification in metagenomes. Using 5390 sequenced microbial genomes, 8 770 321 50-mer strain-specific and 11 736 360 species-specific GSMs were identified for 4088 strains and 2005 species (4933 strains), respectively. The GSMs were first evaluated against mock community metagenomes, recently sequenced genomes and real metagenomes from different body sites, suggesting that the identified GSMs were specific to their targeting genomes. Sensitivity evaluation against synthetic metagenomes with different coverage suggested that 50 GSMs per strain were sufficient to identify most microbial strains with ≥0.25× coverage, and 10% of selected GSMs in a database should be detected for confident positive callings. Application of GSMs identified 45 and 74 microbial strains/species significantly associated with type 2 diabetes patients and obese/lean individuals from corresponding gastrointestinal tract metagenomes, respectively. Our result agreed with previous studies but provided strain-level information. The approach can be directly applied to identify microbial strains/species from raw metagenomes, without the effort of complex data pre-processing.

## INTRODUCTION

Microorganisms can be found in almost every environment of the Earth’s biosphere and are responsible for numerous biological activities including carbon and nitrogen cycling (1), organic contaminant remediation (2–4) and human health and disease. Many human disorders, such as type 2 diabetes (T2D), obesity, dental cavities, cancer and some immune-related diseases, are known to be related with a single or a group of microorganisms (5–11). In addition, different strains within the same species may have completely different impacts on human health, such as *Escherichia coli* O157:H7 (12), which is a highly virulent *E. coli* strain, whereas most other strains in this same species are non-pathogenic. Thus, characterization and identification of microbial strains/species in the environment and individual human hosts is of crucial importance to reveal human–microbial interactions, especially for patients with microbial-mediated disorders. Although different technologies have been developed, the characterization and identification of known microorganisms at strain/species levels remain challenging, mainly due to the lack of high-resolution tools and the extremely diverse nature of microbial communities.

Currently, the most commonly used approach to characterize and identify microorganisms in complex environments is to sequence 16S ribosomal RNA (rRNA) gene amplicons using universally conserved primers (13). However, owing to the high similarity of 16S rRNA gene sequences among different microorganisms, this approach can only confidently identify microorganisms at high taxonomic levels (e.g. genus and family) but not at the species/strain level, although species identification had been attempted in a few studies with less complex communities (14,15). Even at the genus level, resolution problems with 16S rRNA gene sequences have been reported by many investigators (16). Therefore, it is necessary to use other molecular markers to identify and characterize microorganisms at the strain/species level in complex environments.

Owing to the advances in next-generation sequencing (NGS) technologies, shotgun metagenome sequencing, which tries to capture all DNA/RNA information directly from environmental samples, has been widely applied to characterize microbial communities in various environments (17–21), including those of the human body (5,8,9,22). Also, with the efforts of the Human Microbiome Project (23), >5000 sequenced microbial genomes are available as references, making it possible for us to identify and characterize those sequenced microbial strains/species in shotgun metagenomes. However, it is computationally intensive using traditional approaches, such as Basic Local Alignment Search Tool (BLAST) (24) searching or short reads mapping (25) metagenomes against currently sequenced microbial genomes (∼5000 genomes), while assembling them into contigs to reduce data sizes is even more challenging (26). Furthermore, many closely related microbial strains/species share large amounts of genome content, which generates a lot of noise in assigning short reads to references, resulting in ambiguous observations. In addition, sequencing errors, a common issue in NGS techniques (27), may also reduce confidence levels and increase ambiguity in assigning reads to reference genome sequences, especially to genomes of highly similar strains. Therefore, there is an urgent need to develop an approach that can accurately identify microbial strains/species from shotgun metagenomes.

Until recently, efforts have been made to unambiguously classify metagenomic reads into species or higher levels using a reduced set of clade-specific genes (28). However, this approach only incorporates gene coding regions in the genomes, leaving intergenic regions untapped. Moreover, strain-level identification of known microorganisms is still not feasible due to the high conservation of clade-specific genes in closely related strains (≥94% average nucleotide identity) (29).

In this study, we developed a novel *k*-mer-based approach, termed GSMer, to identify genome-specific markers (GSMs) from currently sequenced microbial genomes, which could then be used for accurate strain/species-level identification of microorganisms in metagenomes. GSMer first identifies a set of GSMs for each genome by rapidly and comprehensively searching all regions in the genome sequence and filtering out non-specific sequences. By searching shotgun metagenomes against these GSMs, the presence/absence and/or the relative abundance of each reference strain/species can be determined. In the following, strain-specific GSMs were evaluated against mock community metagenomes, recently sequenced genomes and real metagenomes from different body sites for specificity. Detection limit and true positive calling thresholds were also determined. It was then applied to identify microbial strains/species associated with T2D and obesity from previously published metagenomes.

## MATERIALS AND METHODS

### Data resources

Reference genome sequences (both finished and draft) targeting 5390 microbial strains were downloaded from Human Microbiome Project Data Analysis and Coordination Center (HMPDACC) and NCBI GenBank databases. Because human DNA may be the main contamination in human microbiome studies, human genome sequences were also downloaded and included for GSM selection. Duplicated genome sequences from different sources were binned together according to the organism information in GenBank files. Body site information for human-associated microbial strains/species was obtained from the HMPDACC project catalog.

Four mock community metagenomes consisting of 21 bacterial strains were downloaded from the National Center for Biotechnology Information (NCBI) Sequence Read Archive (SRA) with accession numbers SRR172902, SRR072233, SRR172903 and SRR072232. Among these, two were even mock communities, and two were staggered mock communities. SRA format shotgun metagenomes were converted to FASTA format files using the *sra* toolkit. Converted FASTA format files were then used to identify microbial strains.

Recently sequenced microbial genomes that were not included in GSM identification were downloaded from the JGI IMG Web site (http://img.jgi.doe.gov/cgi-bin/w/main.cgi). A total of 302 finished genomes were downloaded. Body site-specific metagenome raw data were downloaded from the HMPDACC Web site for specificity evaluation of selected GSMs. For each body site, the largest metagenome data set available was selected. Nine bz2 compressed fastq format metagenomes from stool (SRS011084, 15.3 Gb), subgingival plaque (SRS019029, 1.9 Gb), tongue dorsum (SRS011115, 9.9 Gb), right retroauricular crease (SRS020263, 4.2 Gb), palatine tonsils (SRS019126, 4.2 Gb), throat (SRS019127, 2.7 Gb), anterior nares (SRS023847, 462.4 Mb), left retroauricular crease (SRS017849, 4.7 Gb) and posterior fornix (SRS023468, 6.4 Gb) were downloaded.

Raw metagenome data sets targeting T2D/control (8) gut microbiomes were downloaded from NCBI SRA under accession numbers SRA045646 and SRA050230. Obese/lean metagenome raw data (5) were downloaded from NCBI SRA under accession number SRA002775. SRA format shotgun metagenomes were converted to FASTA format files using the *sra* toolkit. Converted FASTA format files were then used to profile disease-associated microbial strains/species.

### Selection of GSMs

First, strain-level non-redundant *k*-mers were generated for all collected microbial strains as well as human genomes. *k*-mers that occurred in two or more bacterial strains were extracted and combined with all *k*-mers of human genomes as a database for stretch filtering. A *k*-mer table was then built by the *meryl* program adopted from the *k*-mer package (30). To ensure high specificity of GSMs and reduce computational cost, *k*-mer sizes ranging from 18 to 20 were used in this study. Second, after transforming GenBank files into FASTA files, each reference genome was split into 50-mer fragments without ambiguous nucleotides (such as Ns and other consensus nucleotides). Thus, for a genome size of L, the number of 50-mer fragments is as much as L-50. Non-redundant 50-mer fragments were identified and kept for further filtering. Third, the *k*-mer-based approach was used to filter out potentially non-specific 50-mer fragments. One significant feature of non-specific DNA fragments is that they share continuous stretch oligonucleotides with their non-targets. Thus, continuous stretch filtering could be used to filter out non-specific 50-mer fragments. Here we used *k*-mer-based strategies for continuous stretch filtering. All *k*-mers in the *k*-mer table were mapped to the 50-mers for each genome by the *mapMers* program (30). Mapped 50-mers were discarded, as they shared *k*-mers with other strains. Finally, remaining 50-mers for each genome were then searched against all microbial genomes and human genomes for further global sequence identity filtering using MEGABLAST (31) to search for the closest non-target sequences and recalculate global sequence identities. All 50-mers that share sequence identity ≥85% between their non-target genomes were discarded. The remaining 50-mers were identified as GSMs.

Species-specific GSMs were identified in a similar way as strain-specific GSMs, but *k*-mer databases were generated at the species level rather than at the strain level. The maximum sequence similarity was calculated between 50-mers and non-target genomes that belong to different species.

To ensure each strain/species has enough GSMs from multiple regions for real applications, a minimum of 50 GSMs/strain was desired. For such a purpose, a progressive *k*-mer filtering approach was used. For example, if <50 GSMs were identified for a strain at a *k*-mer size of 18, the strain would be subject to GSM identification using a *k*-mer size of 19 and/or 20. The same procedure was also applied to identify species-specific GSMs. Microbial strains with <50 GSMs/strain at both strain/species levels with all three *k*-mer sizes were excluded for disease-associated strain/species profiling, although more GSMs might be found at longer *k*-mer sizes.

### Specificity evaluation of GSMs

To evaluate the specificity of identified GSMs with known bacterial genomes, GSMs from all available microbial strains/species (50 GSMs/strain) were searched against the mock community consisting of 21 bacterial genomes using MEGABLAST (31). Only perfect matches between metagenome reads and GSMs were considered as effective hits. The same criteria were used for specificity evaluation against recently sequenced microbial genomes.

To evaluate the specificity of identified GSMs with unsequenced bacterial genomes, GSMs were separated into different groups by the body site from which the microorganisms had been isolated. Body site information for microbial strains was obtained from the HMP DACC Web site. Only strains linked to one body site were selected. Six groups of GSMs were extracted for evaluation, targeting body sites including oral, gastrointestinal tract, airways, skin, urogenital tract and blood. For each body site, 80 strains with >50 GSMs identified were randomly selected. For each randomly selected microbial strain, 50 GSMs were randomly selected, resulting in 24 000 GSMs in total. Metagenomes from different body sites were searched against the selected GSMs using MEGABLAST (31). Only perfect matches between metagenome reads and GSMs were considered as effective hits. It is expected that GSMs targeting microorganisms isolated from one body site will be less likely to be perfectly matched with metagenomes from other distinct body sites because different body sites should host different microbial communities.

### Determining the detection limit and true positive thresholds

Gut GSMs and their targeted genomes were extracted for evaluation. The identification rates between different numbers of GSMs per strain and different sequencing coverage of microorganisms were analyzed. Simulated metagenomes targeting 695 gut microbial genomes were generated by the Grinder program (32), with coverage ranging from 0.01 to 0.75. Paired-end 100-base reads were randomly generated. Randomly selected GSMs with numbers of 1, 5, 10, 25, 50, 100, 200 and 500 GSMs/strain were used for evaluation. The simulated metagenomes were searched against GSMs using MEGABLAST (31) for strain/species identification. Only perfect matches were regarded as effective hits.

### Profiling T2D-/obesity-associated microbial strains

Raw metagenome reads were downloaded and searched against gastrointestinal tract GSMs using the MEGABLAST program (31). Because different microbial strains/species may have different numbers of GSMs, we randomly selected 50 GSMs for each strain for normalization purposes in statistical analysis. Only perfect matches between metagenome reads and GSMs were extracted for statistical analysis. Normalization of BLAST hits profile representing the abundance of microbial strains/species was based on the total number of raw reads and then further normalized to 10 000 000 (Illumina) or 1 000 000 (454) to avoid too small relative abundance values. Student’s *t*-test was applied to evaluate statistical significance of T2D-associated microbial strains/species. Response ratio analysis was used to illustrate obesity-associated microbial strains/species. Benjamini–Hochberg false discovery rate (FDR) analysis was applied to detected microbial strains with ≥5 normalized reads to see how many microbial strains remained significant after *P*-value correction.

## RESULTS

### Selection of strain/species-specific GSMs

**Figure 1.**
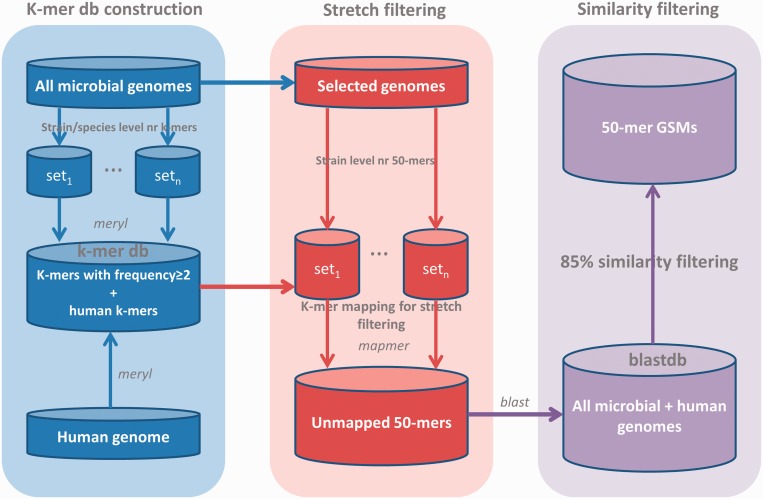
Flowchart of GSM identification processes. First, *k*-mer database (db) construction. *K*-mer db representing k-mers that show up in two or more microbial strains and all human genome *k*-mers were constructed by meryl program. *K*-mer sizes from 18 to 20 were selected. Second, 50-mer GSMs were generated for selected strains/species. GSMs were then mapped with the *k*-mer db, and mapped GSMs were filtered. Third, all GSMs were searched against all microbial genomes by BLAST, and GSMs having 85% identity with non-target GSMs were also filtered.

To our best knowledge, no comparative (meta)genomic tools are currently available to identify genome-specific regions from >5000 microbial genomes. Here we developed a novel approach to identify GSMs of the same length by taking advantage of *k*-mer-based approaches. Two different criteria, including continuous stretch match length and maximum sequence identity with their non-targets, were used to ensure the specificity of GSMs. The whole process of GSM identification was illustrated in the flowchart (Figure 1). Because a clear definition of microbial strains and species is still widely debated, strains and species here were defined based on the NCBI classification system, where the binomial nomenclature part defines a species and the ID followed by the binomial name defines a strain.

We used two different criteria to identify highly specific GSMs. One is that all GSMs should not have a continuous stretch length ≥21-base match with non-target genomes. The other is that all GSMs should not have a sequence identity ≥85% with non-target genomes. To ensure the identified GSMs are highly specific to their target genomes and to reduce the computational time for GSM identification, we started to identify GSMs using a continuous stretch cutoff of 18-mer, then progressively increased the stretch length for genomes without GSMs using the previous stretch length, until 20-mer (Figure 1). The 18-mer starting point was selected for its having relatively large amount (>10 million) of candidate GSMs after *k*-mer continuous stretch filtering, whereas 17-mer stretch filtering only resulted in ≤20 000 GSMs for ≥5000 genomes (Supplementary Figure S1).

**Figure 2.**
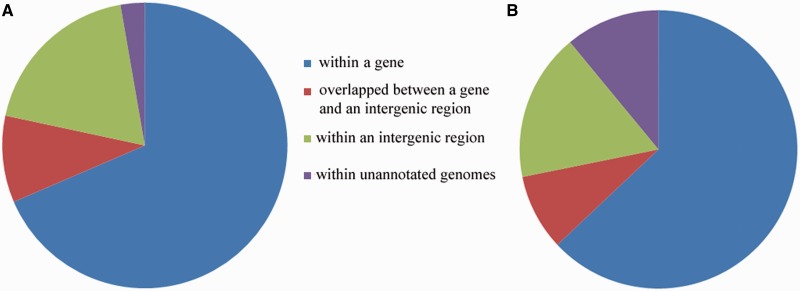
Location of the identified GSMs in the genome. (**A**) strain-specific GSMs; (**B**) species-specific GSMs. Different colors denote different locations in the genome: blue for GSMs within genes, green for GSMs within intergenic regions, red for GSMs overlapped between a gene and an intergenic region and purple for unannotated genomes.

As a result, of the 5390 microbial strains subject to GSM identification, 4088 could have ≥50 strain-specific GSMs identified. Among them, 2548 were identified at the 18-mer stretch length, 1161 at the 19-mer stretch length and 384 at the 20-mer stretch length. A total of 8 770 321 strain-specific GSMs were identified, among which 6 011 103 (68.5%) were located within genes, 1 657 931 (18.9%) within intergenic regions, 861 008 (9.8%) overlapped between gene and intergenic regions and 240 092 (2.7%) were from unannotated genomes (Figure 2A). Considering the ratio of genes and intergenic regions in a typical bacterial genome (∼4.9:1), a higher relative percentage of GSMs was located in or partially in intergenic regions. This also indicated the importance of intergenic regions in bacterial genomes, especially for microbial identification.

GSMs that target multiple strains in the same species were defined as species-specific GSMs. A total of 11 736 360 GSMs targeting 2005 species (4933 strains) were identified. Among them, 1872 species (3219 strains) were identified at an 18-mer size, 198 species (1454 strains) at a 19-mer size and 48 species (260 strains) at a 20-mer size. Approximately 63% (7 391 847) were located within genes, 8.8% (1 037 718) overlapped between a gene and its intergenic regions, 17.2% (2 016 522) were within intergenic regions and 11% (1 290 273) were from unannotated genomes (Figure 2B). This distribution was generally consistent with strain-specific GSMs, suggesting that intergenic regions are important for selecting species-specific GSMs. To select GSMs for the remaining microbial strains without GSMs using the above criteria, modified strategies such as longer stretch length and/or relaxed identity cutoffs could be used.

### Specificity evaluation with mock community metagenomes

To check the specificity of GSMs with currently sequenced genomes, we first evaluated the selected GSMs against a mock microbial community consisting of 21 bacterial species (23), of which 16 species had GSMs available. As a result, all 16 (100%) bacterial strains were identified without false-positive results for the two ‘even-distributed’ mock community data sets sequenced by Illumina and 454 (SRR172902 and SRR072233). Twelve (75%) and 14 (87.5%) true-positive findings were identified for the two staggered mock community data sets—SRR172903 and SRR072232, respectively. False-negative identification in the staggered mock communities was due to the low coverage of these strains (Supplementary Table S1). Three false-positive findings were found in the data set SRR172903 with only one mapped read for each strain. Of these, two belonged to closely related strains at the same species; thus, it might be caused by the incomplete sequencing of these strains or contamination (Supplementary Table S1). However, these false-positive results could be effectively removed if a cutoff of identified reads number (e.g. 5) and/or mapped GSM number (e.g. 5) were used.

### Specificity evaluation against recently sequenced genomes and body site-specific metagenomes

**Figure 3.**
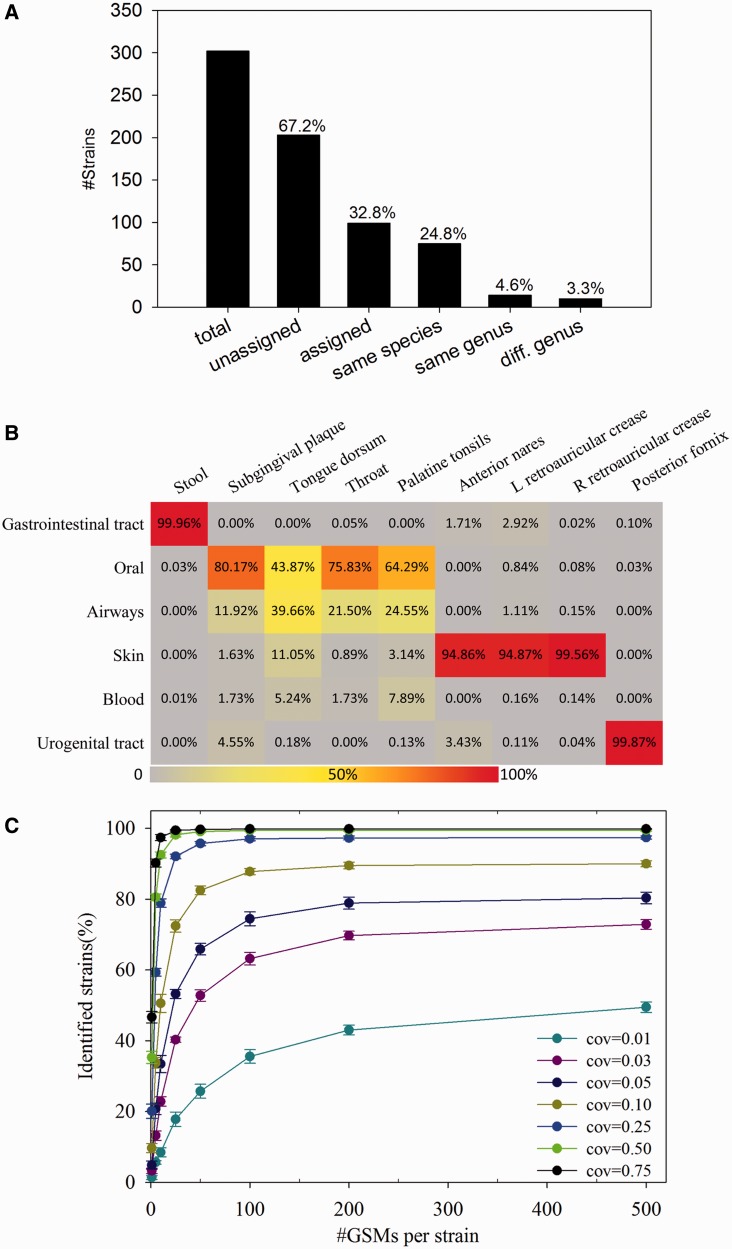
Specificity and sensitivity evaluation of identified GSMs. (**A**) Specificity evaluation against recently sequenced genomes. A total of 302 genomes were collected. (**B**) Specificity evaluation of GSMs targeting microorganisms isolated from different body sites using raw metagenomes reads. GSMs targeting six different body sites (gastrointestinal tract, oral, airways, skin, blood and urogenital tract) were searched with metagenomes from nine different body sites (stool, subgingival plaque, tongue dorsum, throat, palatine tonsils, anterior nares, left retroauricular crease, right retroauricular crease and posterior fornix) using MEGABLAST. Numbers denote the percentages of MEGABLAST hits, with GSMs targeting each body site. (**C**) Sensitivity evaluation of GSMs using simulated metagenomes from 695 guts microbial strains. Simulated metagenomes at seven different coverages (0.01, 0.03, 0.05, 0.1, 0.25, 0.5 and 0.75) were searched against different number of GSMs per strain (1, 5, 10, 25, 50, 100, 200 and 500). The percentages of identified microbial strains were analyzed.

Another question is how specific the GSMs are to unsequenced genomes. This is also critical for true-positive callings from metagenomes because the majority of microbial genomes are not yet sequenced, although >5390 microbial strains were used for GSM identification. To evaluate the specificity of GSMs with unsequenced genomes, we collected 302 finished genomes that were recently sequenced (not included in the GSM target strains) and searched them against strain-specific GSMs. A total of 203 (67.2%) genomes were not assigned to any genomes (Figure 3A). Of the 99 (32.8%) genomes assigned to the strains in the GSM database, 75 (24.8%) were assigned to closely related strains in the same species, 14 (4.6%) to the same genus but different species and only 10 (3.3%) were assigned to different genera (Figure 3A). This suggests that the GSMs identified in this study are even highly specific to unsequenced microbial genomes.

In addition, we also performed an alternative evaluation to verify the specificity of GSMs, which was less rigorous but still illustrative. In this test, we hypothesized that microorganisms isolated from one body site are less likely to be found in another distinctly different body site, based on current studies that different body sites host different microbial communities (33). Body site information for microbial strains was obtained from the HMP DACC Web site. GSMs of microbial strains linked with only one of the major six body sites were extracted, although the possibility existed that some strains may also be found in other body sites. Selected GSMs were searched with raw metagenome data from different body sites. It was expected that far fewer hits could be found in other body sites than the particular body site that the selected strains were isolated from. As a result, of the six groups of GSMs targeting different body sites, three are highly specific to their corresponding body sites, and one (blood GSMs) rarely had any hits because it did not have any corresponding metagenomes (Figure 3B). For example, gut GSMs were mainly targeted by stool metagenomes (99.96%); skin GSMs were mainly targeted by metagenomes from anterior nares (94.86%), left retroauricular crease (94.87%) and right retroauricular crease (99.56%); urogenital tract GSMs were mainly targeted by posterior fornix metagenome (99.87%) (Figure 3B). The only exception was that a relatively high number of oral metagenomes, including tongue dorsum (39.66%), throat (21.50%) and palatine tonsils (24.55%), were hit by GSMs targeting microorganisms isolated from airways. Because these oral sites are so closely located and connected with airways and share similar physiological and functional properties, it is possible for some microorganisms to co-occur in different body sites. This was also evidenced by previous studies that microbes from oral sites, such as tongue, tonsils, throat, saliva and gingival plaques, contribute to the colonization in the airways for their important overlap between the upper segments of the digestive and respiratory segments (34). In addition, low BLAST hit numbers were observed between these oral metagenomes and GSMs targeting gut, skin, blood or urogenital microorganisms, confirming the strong possibility that the hits between oral metagenomes and airway GSMs resulted from their sharing some microorganisms.

These results also suggested that the identified GSMs were highly specific to their targeted microorganisms.

### Determining the detection limit and true positive calling thresholds for microbial identification using GSMs

Detection limit (sensitivity) is another important issue to identify microbial strains/species from short metagenome sequences in complex environments. There are two major questions associated with sensitivity: (i) At what sequencing coverage could the microbial genome be identified by GSMs? (ii) How many GSMs are required for effective identification of microbial strains/species? To answer these two questions, simulated metagenomes with different degrees of genome coverage were generated from sequenced genomes and then used to determine how many GSMs could be identified. Of the 695 gut microbial genomes subjected to evaluation, ∼40% could be detected at 0.01× sequencing coverage level when ≥100 GSMs/strain were used. The value increased to ∼90% with 0.1× sequencing coverage and nearly 100% for 0.25× sequencing coverage with ≥50 GSMs/strain (Figure 3C), and the trend became saturated at 200 GSMs/strain. Overall, our results suggested that the minimum required GSMs/strain for low-coverage (≤0.25×) sequence data is 100, and 50 for reasonable sequence coverage (≥0.25×) sequence data. However, it should be noted reasonable sequencing coverage of a metagenome is necessary for any methods to identify its specific members, especially at the strain/species level.

Another issue related with microbial identification is the threshold for positive calling of an identified strain or species. To confidently identify a microbial strain/species in a metagenome, a proper threshold of mapped GSMs is necessary. Hence, we examined the distribution of mapped GSM numbers for the simulated metagenomes when 50 and 100 GSMs/strain were used (Supplementary Figure S2). With 50 GSMs/strain, >94% of microbial strains with 0.5× and 0.75× sequencing coverage were identified with 6–50 GSMs (Supplementary Figure S2B and C), and with 100 GSMs/strain, >95% were identified with 11–100 GSMs (Supplementary Figure S2E and F). Even at 0.25× sequencing coverage, >75% microbial strains were identified with ≥6 GSMs and ≥11 GSMs when 50 and 100 GSMs/strain were used, respectively (Supplementary Figure S2A and D). In addition, it was found in the specificity evaluation section that ∼82% non-specific identifications in metagenomes from different body sites were with ≤5 GSMs (∼2 GSMs/strain). These results suggested that a 10% threshold cutoff (e.g. 5–10 GSMs per strain/species) of the number of selected GSMs could be recommended for positive callings. However, to detect low-coverage microbial strains/species, a lower cutoff could be used with the potential trade-off of increased false-positive identifications.

### Comparison with other approaches

To our best knowledge, no approaches are yet available to perform strain-level analysis of shotgun metagenomes. Here we performed species-level analysis for synthetic shotgun metagenomes generated from the 302 recently sequenced microbial genomes, and compared the results with the current state-of-the-art MetaPhlAn. Of the 192 microbial species targeted by the synthetic metagenome, MetaPhlAn made 68 and 69 true-positive identifications and 38 and 41 false-positive identifications at the sensitive and very sensitive modes, respectively. When ≥5 and ≥1 mapped GSMs were used as cutoffs, our GSMer approach showed slightly fewer true-positive identifications (58 and 62) but much fewer false-positive identifications (16 and 21) (Supplementary Figure S3). Such differences in true-positive and false-positive identifications should be due to the higher specificity nature of identified GSMs. For both GSMer and MetaPhlAn, about two-thirds of the microbial species targeted by the recently sequenced genomes were not identified, indicating that both GSMer and MetaPhlAn to be specific tools for sequenced microbial genomes. This also indicated that such limitations in identifying mainly known microbial strains/species could be a common issue for strain/species-level taxonomic identifiers. To increase the ability of identifying more microbial strains/species, more newly sequenced microbial genomes need to be included.

### Metagenomic profiling of T2D-associated microbial strains/species

To evaluate whether our selected GSMs could be applied to identify disease-associated microbial strains/species in the human body, GSMs targeting human gut microorganisms were searched with raw metagenome data from 345 Chinese individuals, with 174 healthy people and 171 diagnosed with T2D (8). The previous study with these metagenomes identified 47 T2D-associated metagenomic linkage groups, of which 17 were assigned to known bacterial species, 8 to genera, 2 to families and 1 to order (8). Thus, it makes us possible to judge the consistency of our results by comparing with this previous study.

With 50 GSMs per strain, 379 microbial strains and 11 species representing 66 strains were found to be present in at least one individual. A total of 45 microbial strains/species were identified to be significantly (*P* ≤ 0.05) related with T2D patients, among which 22 had average normalized hits ≥5. After Benjamini–Hochberg FDR correction, six strains remained to be significant (Table 1). Because the FDR procedure is closely related with the number of detected microbial strains and all detected microbial strains could be considered independent and uncorrelated, all 22 potential T2D-associated microbial strains with Student’s *t*-test *P* ≤ 0.05 without FDR correction were analyzed here. Of them, 14 were enriched in T2D patients, whereas the remaining 8 were enriched in healthy individuals (Table 1). Further literature mining showed that many of the T2D-enriched microbial strains/species were previously identified as potential opportunistic pathogens, such as *Bacteroides caccae* ATCC 43185 (35), *Clostridium bolteae* ATCC BAA-613 (36), *Escherichia coli* DEC6E or not yet well-characterized microbial strains that are distinct from currently recognized strains such as those named *Alistipes* sp., *Bacteroides* sp., *Parabacteroides* sp. and *Subdoligranulum* sp. In addition, the mucin-degrading strain *Akkermansia muciniphila* ATCC BAA-835 was also found to be significantly enriched in T2D patients, which was also observed in the previous study (8). In contrast, most microbial strains enriched in healthy individuals belong to butyrate-producing bacteria, such as *Clostridiales bacterium* SS3/4, *Eubacterium rectale* ATCC 33656, *E. rectale* DSM 17629, *Faecalibacterium cf. prausnitzii* KLE1255, *Roseburia intestinalis* XB6B4 and *Roseburia inulinivorans* DSM 16841. Two *Prevotella* strains, *Prevotella copri* DSM 18205 and *Prevotella stercorea* DSM 18206, which were reported to be highly associated with carbohydrate consumption (37), were also found to be significantly (*P* < 0.05) enriched in healthy individuals. These results agreed with previous results based on metagenome-wide association studies (8), but provided more detailed information at the strain level.

**Table 1.** The list of microbial strains significantly associated with T2D patients with mean normalized hits ≥5 in treatment/control

Strain	Number of mean normalized hits ± SDOM		*P*-value	*P*-value after FDR correction
	Control	Treatment		
T2D-enriched				
* Akkermansia muciniphila* ATCC BAA-835	4.79 ± 1.72	18.12 ± 4.58	0.0065	0.07
* Alistipes indistinctus* YIT 12060	3.60 ± 1.02	8.40 ± 1.84	0.0222	0.15
* Alistipes* sp. HGB5	3.43 ± 0.40	6.58 ± 1.14	0.0090	0.06
* Bacteroides caccae* ATCC 43185	29.88 ± 3.39	56.74 ± 8.38	0.0030	0.04
* Bacteroides cellulosilyticus* DSM 14838	9.27 ± 1.90	17.08 ± 3.31	0.0405	0.17
* Bacteroides* sp. 2_1_16	2.87 ± 0.57	5.90 ± 1.41	0.0454	0.21
* Bacteroides* sp. 2_1_33B	4.20 ± 0.55	8.84 ± 2.00	0.0247	0.13
* Bacteroides* sp. 20_3	15.18 ± 1.96	33.66 ± 4.71	0.0003	0.02
* Bacteroides* sp. D22	4.03 ± 0.54	6.18 ± 0.78	0.0245	0.15
* Clostridium bolteae* ATCC BAA-613	3.28 ± 0.53	22.50 ± 9.04	0.0330	0.15
* Escherichia coli* DEC6E	1.27 ± 0.42	5.47 ± 1.85	0.0261	0.16
* Lachnospiraceae bacterium*	17.93 ± 2.34	26.61 ± 3.74	0.0492	0.15
* Parabacteroides* sp. D13	4.51 ± 0.93	8.04 ± 1.05	0.0124	0.06
* Subdoligranulum *sp. 4_3_54A2FAA	2.04 ± 0.31	6.65 ± 1.49	0.0025	0.05
Control-enriched				
* Clostridiales bacterium* SS3/4	10.05 ± 0.85	7.35 ± 0.92	0.0318	0.19
* Eubacterium rectale* ATCC 33656	5.58 ± 1.15	2.91 ± 0.45	0.0319	0.14
* E. rectale* DSM 17629	7.07 ± 1.05	3.50 ± 0.52	0.0026	0.05
* Faecalibacterium cf. prausnitzii* KLE1255	20.46 ± 2.30	12.75 ± 2.03	0.0124	0.14
* Prevotella copri* DSM 18205	204.12 ± 33.5	106.57 ± 22.4	0.0164	0.11
* Prevotella stercorea* DSM 18206	58.41 ± 14.61	14.00 ± 5.77	0.0052	0.04
* Roseburia intestinalis* XB6B4	15.78 ± 2.14	7.30 ± 1.30	0.0008	0.04
* Roseburia inulinivorans* DSM 16841	34.08 ± 4.61	21.76 ± 3.59	0.0360	0.18

### Metagenomic profiling of obesity-associated microbial strains/species

Gut GSMs were then applied to identify obesity-associated microbial strains/species in human gut micorbiomes by searching gut GSMs with metagenomes from 18 individuals, of whom 9 were diagnosed as obese, and the rest were lean/overweight (5). The comparison (i.e. obese versus lean/overweight) was carried out in the same manner as in the original study (5). The previous study found an increased abundance of Actinobacteria and a decreased abundance of *Bacteroides* in obese individuals, but strain/species level-identification of microorganisms associated with obesity was not carried out. Here we intend to identify microbial strains/species associated with obesity, and at the same time to evaluate our results with this previous study by summarizing our data at the phylum level.

**Figure 4.**
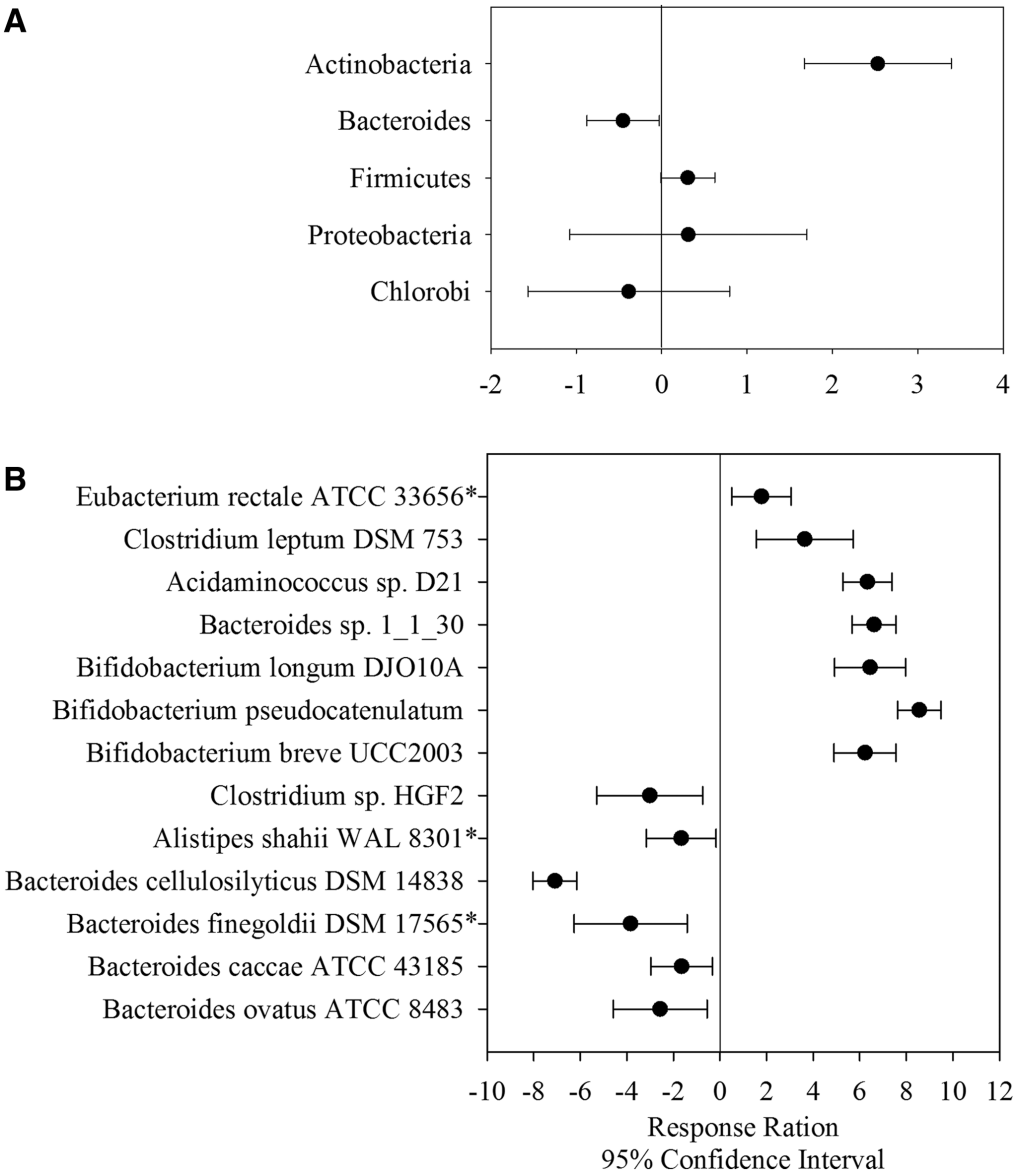
Response ratio analysis of obese/lean-associated microorganisms at the phylum (**A**) and strain/species level (**B**). For strain/species-level analysis, only significantly associated ones with normalized hit number ≥5 were displayed. Asterisks refer to microbial strains that did NOT pass Benjamini–Hochberg FDR analysis at a corrected *P*-value cutoff of 0.05.

As a result, 159 microbial strains/species were detected in at least one sample in the study. Response ratio analysis showed the relative abundance changes of microbial strains/species between obese and lean/overweight individuals at the 95% confidence interval level. To evaluate whether our results were consistent with the previous one, we first summarized and analyzed the relative abundances of microbial strains/species at the phylum level. A significant lower abundance of *Bacteroides* and higher abundance of Actinobacteria were found in obese individuals than those in lean/overweight individuals, whereas no significant changes were observed for microbial phyla such as Firmicutes, *Proteobacteria* and *Chlorobi* between those two groups (Figure 4A). The results were consistent with the previous report using the whole metagenome BLAST searching approach (5). We then analyzed the relative abundances of microorganisms at the strain/species level. Relative abundances of 74 strains/species were identified to be significantly (*P* < 0.05) changed in obese/lean individuals. Among these, 13 were found to have an average normalized BLAST hit number ≥ 5 in obese or lean/overweight individuals. Only three did not pass Benjamini–Hochberg FDR analysis at corrected *P*-value cutoff of 0.05 (Figure 4B). Of these, six microbial strains were enriched in lean/overweight individuals with five (*Bacteroides cellulosilyticus* DSM 14838, *Bacteroides finegoldii* DSM 17565, *B. caccae* ATCC 43185, *Bacteroides ovatus* ATCC 8483 and *Alistipes shahii* WAL 8301) in the *Bacteroides/Chlorobi* group and one (*Clostridium* sp*.* HGF2) in Firmicutes. Of the seven obesity-enriched microbial strains/species, three (*Bifidobacterium breve* UCC2003, *Bifidobacterium pseudocatenulatum* and *Bifidobacterium longum* DJO10A) belonged to Actinobacteria, three (*Acidaminococcus* sp*.* D21, *Clostridium leptum* DSM 753 and *E. rectale* ATCC 33656) were Firmicutes and one (*Bacteroides sp.* 1_1_30) was *Bacteroides*. Literature search suggests that most microbial strain/species enriched in lean/overweight individuals exhibited potential antibiotic/anti-anaerobic-pathogen resistance abilities (38,39), while obesity-enriched microorganisms were mostly probiotics (40,41) and butyrate-producing microorganisms (42,43). These results provided new insights for a better understanding of microorganisms associated with obesity at the strain level.

## DISCUSSION

Comparing with other approaches such as BLAST searching against whole genomes for strain/species identification, our approach reduced the searching database to ∼0.05% of the whole genomes and minimized noise in strain-level microbial identification. Noise could be introduced when searching metagenomes against whole reference genomes. First, sequencing errors or low-quality bases are a common issue in NGS technology (27). Reduced sequence identity was reported when aligning such error-prone reads against long reference genomes, while the issue can be effectively avoided when searching against short GSMs. Second, the majority of genome content is similar among closely related strains, whereas only small portions are strain/species-specific. Ambiguous assignment of reads to reference genomes in such cases unavoidably introduces great noise for statistical analysis in comparative studies, resulting in ambiguous observations. Because GSMs were extracted from genome-specific regions, reads not specific to these regions will not be assigned, resulting in more confident microbial identification and statistical analysis.

Specificity is the most important issue for GSM identification. Non-specific GSMs could lead to inaccurate and ambiguous results for strain/species identification in metagenomes. To ensure highly specific GSMs, several progressive steps were applied. First, >5390 sequenced microbial genomes as well as human genome were used to build *k*-mer databases that feature *k*-mers presenting in two or more genomes, which ensures a comprehensive data source in the beginning. Second, GSMs that can be mapped to any *k*-mers in the databases were discarded, ensuring all remaining GSMs do not have any continuous stretch of *k*-mers (18≤ *k* ≤20) with non-target genomes. Third, GSMs that share a sequence identity of 85% to their non-target genomes were also discarded, further assuring the specificity of identified GSMs. Fourth, all GSMs were identified as 50-mers, which are shorter than current NGS reads length and can be used for ‘perfect matching’ identification of microbial strains. Finally, our evaluation of GSMs against recently sequenced microbial genomes and metagenomes from different body sites showed they are highly specific.

Sensitivity is another important issue in using GSMs for microbial identification. On one hand, the selected GSMs could be from specific regions of one genome (strain-specific GSMs) or multiple genomes (species-specific GSMs), but not all GSMs could be covered by shotgun metagenome sequences, resulting in false-negative detections. On the other hand, most microorganisms in the environment are not sequenced yet, so those incomplete and/or unsequenced genomes may also contain some GSMs identical to those sequenced genomes, leading to false-positive detections. Thus, an appropriate number of GSMs and threshold should be determined for confident positive callings of identified microbial strains/species. Our evaluation using simulated metagenomes suggested that a minimum of 50 GSMs per strain and a 10% cutoff for mapped GSMs shall be used for positive callings for most microbial strains at ≥0.25× sequencing coverage.

A large number of GSMs were identified from intergenic regions for both strain and species-specific GSMs. Intergenic regions comprise ∼15% of bacterial genomes (44), and are usually discarded from data analysis in metagenomes at the gene prediction step. The current interest in intergenic regions is focused on exploring novel functional units such as small RNAs, small ORFs, pseudogenes, transposons, integrase binding sites and repeat elements (45). Our results showed that intergenic regions also contributed heavily to GSMs, suggesting their important roles in identifying microbial strains/species. Thus, here we recommended that gene-prediction-free metagenomes should be used for strain/species identification, and the importance of bacterial intergenic regions should be further recognized.

T2D is a complex system level disorder influenced by both genetic and environmental factors (46,47), as well as the gut microbiome (8,48). Previous studies have suggested significantly different gut microbiome compositions between T2D patients and healthy individuals (6), as well as a group of microbes significantly associated with T2D patients (8). By searching metagenome raw reads against 34 750 randomly selected GSMs targeting 695 gut microbial strains, we identified 390 microbial strains/species present in at least one individual. The 45 microbial strains/species significantly associated with T2D were highly consistent with the previous metagenome-wide association study, showing that more ‘bad’ microbes were enriched in T2D patients, while more ‘good’ microbes were enriched in healthy individuals. Comparing with the metagenomic linkage group approach, one shortage of our approach is that only sequenced microbial strains/species can be identified, but disease-associated markers from unknown species are not targeted. However, this problem can be solved as more reference genomes are sequenced.

Obesity is a genetically, environmentally and microbially associated energy imbalance disorder in the human body. Studies implementing 16S rRNA sequencing as well as shotgun metagenomes demonstrated significant links between the relative abundances of Actinobacteria, *Bacteroides*, Firmicutes and obese hosts (5,39), such as increased Actinobacteria abundance and Firmicutes/*Bacteroides* ratio, and decreased *Bacteroides*. Our phylum level analysis of identified microbial strains using GSMs agreed with these findings. Intriguingly, unlike the increased opportunistic pathogenic microbes in T2D patients, our strain-level analysis showed obese and lean/overweight individuals were associated with different groups of ‘good’ microbes: higher probiotics and butyrate-producing bacteria in obese individuals for maintaining a healthy gut microbiome (49) and providing energy source for intestinal epithelial cells (50), and higher antibiotic/anti-anaerobic-pathogen bacteria in lean/overweight individuals. These observations suggested that both obese and lean/overweight individuals host a healthy gut microbiota, but were enriched by different groups of microbes that harbor different functions.

Both species- and strain-specific GSMs were provided in this study, for the purposes of microbial species and strain identification in metagenomes. Because the majority of currently sequenced microbial strains were covered by the identified GSMs, we expect the method could also be applied to analyze metagenomes from other environments, with the aim to identify sequenced microbial strains/species. However, potential problems may exist, especially for complex microbial communities from environments with limited coverage of reference genomes such as soil, for which the majority of microbial strains are still not yet cultivated and most microbial strains are sequenced with low coverage owing to the extremely high diversity of the community. Such problems would lead to higher false-positive findings and low number of confidently identified microbial strains/species. Thus, we recommend mainly using the developed GSMer approach for metagenomes with good coverage of reference genomes such as human microbiome. For complex metagenomes without good coverage of reference genomes, high-level taxonomic classifiers [e.g. MEGAN (51)] should be used for comprehensive data analysis, while high-resolution identifiers like GSMer can be used to identify known microbial strains/species with ≥0.25× coverage. Even with species-specific GSMs, it seems that the majority of novel microbial strains/species still cannot be identified by such high-resolution taxonomic identifiers, which is also the same case for MetaPhlAn, although some of them could be assigned to their nearest neighbors. With more novel microbial species/strains being isolated and sequenced, we expect that such problems could be effectively solved by incorporating more novel microbial genomes.

In conclusion, the GSMer approach we developed here can be used for direct, rapid and accurate identification of microorganisms at the strain/species level from metagenomes, providing a general tool for analysis of metagenome sequencing data. This approach does not require any efforts for preprocessing of huge deluges of reads, including quality trimming, gene prediction, metagenome assembly and protein-domain matching. In addition, with the advantage of directly taking raw reads, it has the potential to detect microbial strains/species present in low abundances, which are hardly assembled. Although only 50-mer GSMs with strict parameters were identified and evaluated here, longer GSMs are also supported by the approach with more relaxed parameters. In addition, both gene and intergenic regions were used for GSM selection, expanding the detection ability of microbial strain/species. With more reference genomes being sequenced owing to the progress of HMP project (52,53), strain/species level identification of microorganisms is highly demanded, such as clinical diagnosis for patients with microbial-related disorders. Our approach provides a great potential in solving such problems. By integrating such small database with NGS platforms, instant detection of microbial strains/species is also possible. When applied properly, the method can also be used to select probes for microbial ecological microarrays, which also faces great challenges with huge amount of sequences available.

## AVAILABILITY

All source code for GSMer and testing data sets as well as identified strain/species-specific GSMs could be found at https://github.com/qichao1984/GSMer and http://ieg.ou.edu/GSMer. A semiannual update to cover more newly sequenced genomes is projected. A full list of 50-mer strain/species-specific GSMs identified for all microbial strains can also be downloaded at the above Web site.

## SUPPLEMENTARY DATA

Supplementary Data are available at NAR Online.

Supplementary Data
