# Supplementary material for: Strain/species identification in metagenomes using genome-specific markers
Source: Nucleic Acids Res. 2014 Feb 12;42(8):e67. doi: 10.1093/nar/gku138 (PMC4005670; doi:10.1093/nar/gku138)
Supplement: Supplementary Data [file supp_42_8_e67__index.html]

Strain/species identification in metagenomes using genome-specific markers — Strain/species identification in metagenomes using genome-specific markers — Supplementary Data 

# Strain/species identification in metagenomes using genome-specific markers

## Supplementary Data

files

**Files in this Data Supplement:**

- Supplementary Data - docx file
