# Supplementary material for: Strain/species identification in metagenomes using genome-specific markers
Source: Nucleic Acids Res. 2014 Feb 12;42(8):e67. doi: 10.1093/nar/gku138 (PMC4005670; doi:10.1093/nar/gku138)

Table S1. Summary of mapped reads and GSMs of mock community metagenomes. Red denote false positives.

| **Accession** | **Strain Name** | **#reads mapped to genome** | **#reads mapped to GSMs** | **#mapped GSMs** |
| --- | --- | --- | --- | --- |
| SRR172902  (Even mock community, Illumina 75bp) | Streptococcus agalactiae 2603V/R | 6104 | 1 | 5 |
|  | Streptococcus mutans UA159 | 113260 | 38 | 43 |
|  | Bacillus cereus ATCC 10987 | 52022 | 2 | 5 |
|  | Actinomyces odontolyticus ATCC 17982 | 154918 | 65 | 37 |
|  | Bacteroides vulgatus ATCC 8482 | 607992 | 98 | 46 |
|  | Acinetobacter baumannii ATCC 17978 | 787612 | 79 | 48 |
|  | Clostridium beijerinckii NCIMB 8052 | 243616 | 32 | 28 |
|  | Deinococcus radiodurans R1 | 2492460 | 802 | 50 |
|  | Enterococcus faecalis OG1RF | 83409 | 30 | 21 |
|  | Lactobacillus gasseri ATCC 33323 | 1771 | 2 | 3 |
|  | Listeria monocytogenes EGD-e | 123554 | 34 | 28 |
|  | Methanobrevibacter smithii ATCC 35061 | 44131 | 22 | 23 |
|  | Pseudomonas aeruginosa PAO1 | 47377 | 6 | 17 |
|  | Rhodobacter sphaeroides 2.4.1 | 211977 | 68 | 39 |
|  | Staphylococcus aureus subsp. aureus USA300_TCH959 | 209230 | 19 | 21 |
|  | Staphylococcus epidermidis ATCC 12228 | 330608 | 423 | 48 |
| SRR072233  (Even mock community, 454 shotgun) | Streptococcus agalactiae 2603V/R | 1227 | 3 | 4 |
|  | Streptococcus mutans UA159 | 22469 | 18 | 36 |
|  | Bacillus cereus ATCC 10987 | 10752 | 9 | 12 |
|  | Actinomyces odontolyticus ATCC 17982 | 34654 | 214 | 48 |
|  | Bacteroides vulgatus ATCC 8482 | 122608 | 266 | 50 |
|  | Acinetobacter baumannii ATCC 17978 | 174655 | 269 | 50 |
|  | Clostridium beijerinckii NCIMB 8052 | 45573 | 99 | 39 |
|  | Deinococcus radiodurans R1 | 524593 | 2511 | 47 |
|  | Enterococcus faecalis OG1RF | 17847 | 63 | 37 |
|  | Lactobacillus gasseri ATCC 33323 | 391 | 2 | 2 |
|  | Listeria monocytogenes EGD-e | 28108 | 86 | 45 |
|  | Methanobrevibacter smithii ATCC 35061 | 8128 | 54 | 30 |
|  | Pseudomonas aeruginosa PAO1 | 10460 | 15 | 20 |
|  | Rhodobacter sphaeroides 2.4.1 | 34127 | 186 | 47 |
|  | Staphylococcus aureus subsp. aureus USA300_TCH959 | 39582 | 60 | 27 |
|  | Staphylococcus epidermidis ATCC 12228 | 68187 | 746 | 49 |
| SRR172903  (Staggered mock community, Illumina 75bp ) | Streptococcus agalactiae 2603V/R | 38388 | 6 | 15 |
|  | Streptococcus mutans UA159 | 625898 | 134 | 50 |
|  | Bacillus cereus ATCC 10987 | 24505 | 1 | 4 |
|  | Actinomyces odontolyticus ATCC 17982 | 786 | 0 | 0 |
|  | Bacteroides vulgatus ATCC 8482 | 1858 | 0 | 0 |
|  | Acinetobacter baumannii ATCC 17978 | 35169 | 1 | 1 |
|  | Clostridium beijerinckii NCIMB 8052 | 97505 | 16 | 16 |
|  | Deinococcus radiodurans R1 | 22254 | 4 | 5 |
|  | Enterococcus faecalis OG1RF | 899 | 0 | 0 |
|  | Lactobacillus gasseri ATCC 33323 | 582 | 0 | 0 |
|  | Listeria monocytogenes EGD-e | 6285 | 1 | 1 |
|  | Methanobrevibacter smithii ATCC 35061 | 307412 | 111 | 49 |
|  | Pseudomonas aeruginosa PAO1 | 200837 | 7 | 11 |
|  | Rhodobacter sphaeroides 2.4.1 | 2069571 | 387 | 50 |
|  | Staphylococcus aureus subsp. aureus USA300_TCH959 | 1838375 | 122 | 28 |
|  | Staphylococcus epidermidis ATCC 12228 | 1732754 | 1836 | 50 |
|  | Streptococcus pneumoniae SP3-BS71 |  | 1 | 7 |
|  | Mycobacterium tuberculosis H37Ra |  | 1 | 2 |
|  | Staphylococcus aureus subsp. aureus TCH130 |  | 1 | 4 |
| SRR072232  (Staggered mock community, 454 shotgun) | Streptococcus agalactiae 2603V/R | 6712 | 5 | 16 |
|  | Streptococcus mutans UA159 | 106100 | 138 | 50 |
|  | Bacillus cereus ATCC 10987 | 4146 | 3 | 5 |
|  | Actinomyces odontolyticus ATCC 17982 | 138 | 1 | 3 |
|  | Bacteroides vulgatus ATCC 8482 | 324 | 1 | 2 |
|  | Acinetobacter baumannii ATCC 17978 | 6089 | 14 | 35 |
|  | Clostridium beijerinckii NCIMB 8052 | 18661 | 40 | 22 |
|  | Deinococcus radiodurans R1 | 2999 | 20 | 15 |
|  | Enterococcus faecalis OG1RF | 132 | 0 | 0 |
|  | Lactobacillus gasseri ATCC 33323 | 106 | 0 | 0 |
|  | Listeria monocytogenes EGD-e | 1009 | 4 | 5 |
|  | Methanobrevibacter smithii ATCC 35061 | 53719 | 331 | 49 |
|  | Pseudomonas aeruginosa PAO1 | 29446 | 61 | 50 |
|  | Rhodobacter sphaeroides 2.4.1 | 213549 | 1327 | 50 |
|  | Staphylococcus aureus subsp. aureus USA300_TCH959 | 309205 | 362 | 28 |
|  | Staphylococcus epidermidis ATCC 12228 | 310448 | 4114 | 50 |

**Fig. S1** Number of candidate GSMs when different k-mer sizes were used for continuous stretch filtering.


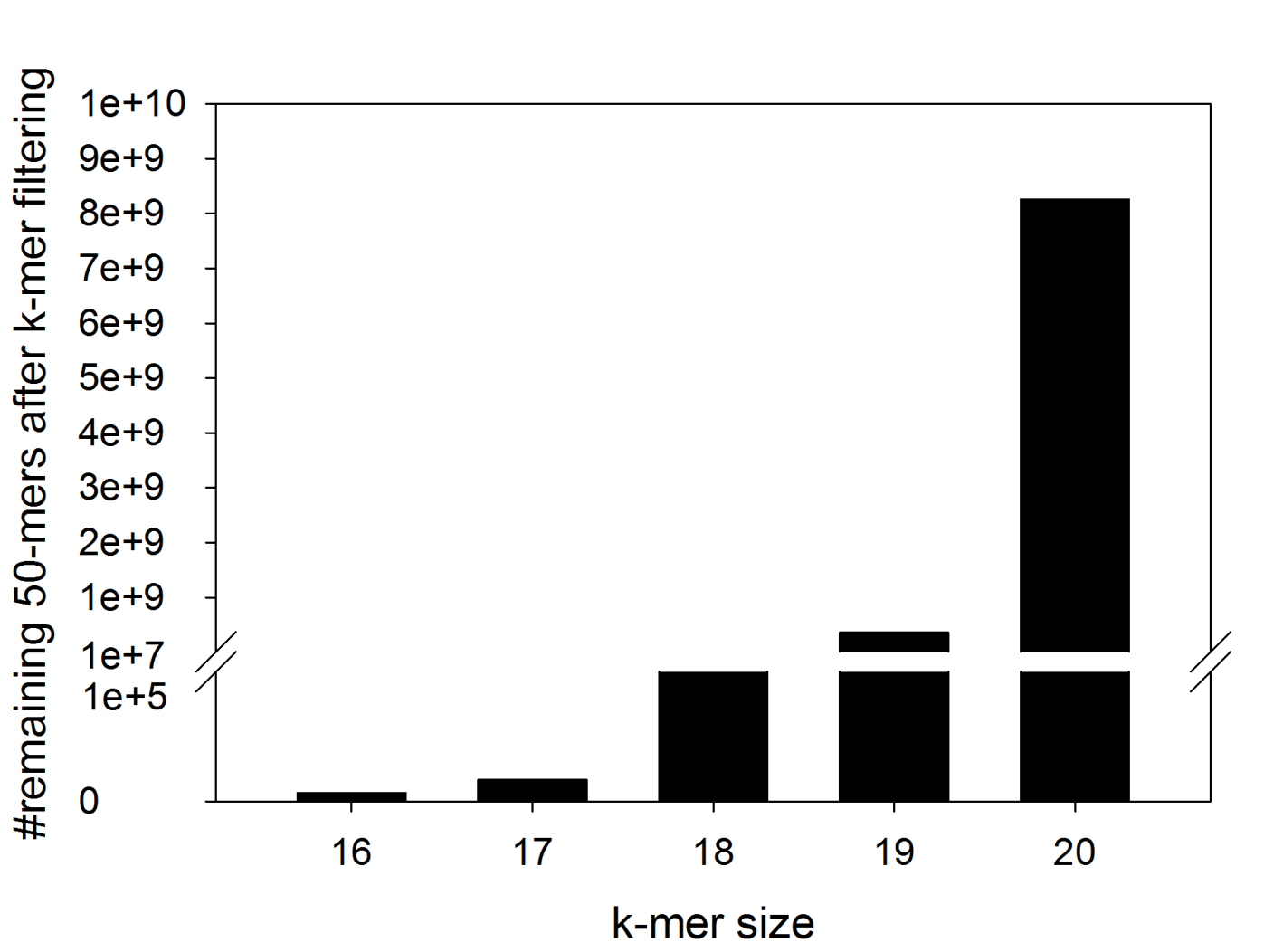


**Fig. S2** Distribution of mapped GSM numbers to simulated metagenomes at sequencing coverage of 0.25, 0.5 and 0.75 with 50 and 100 GSMs/strain used.


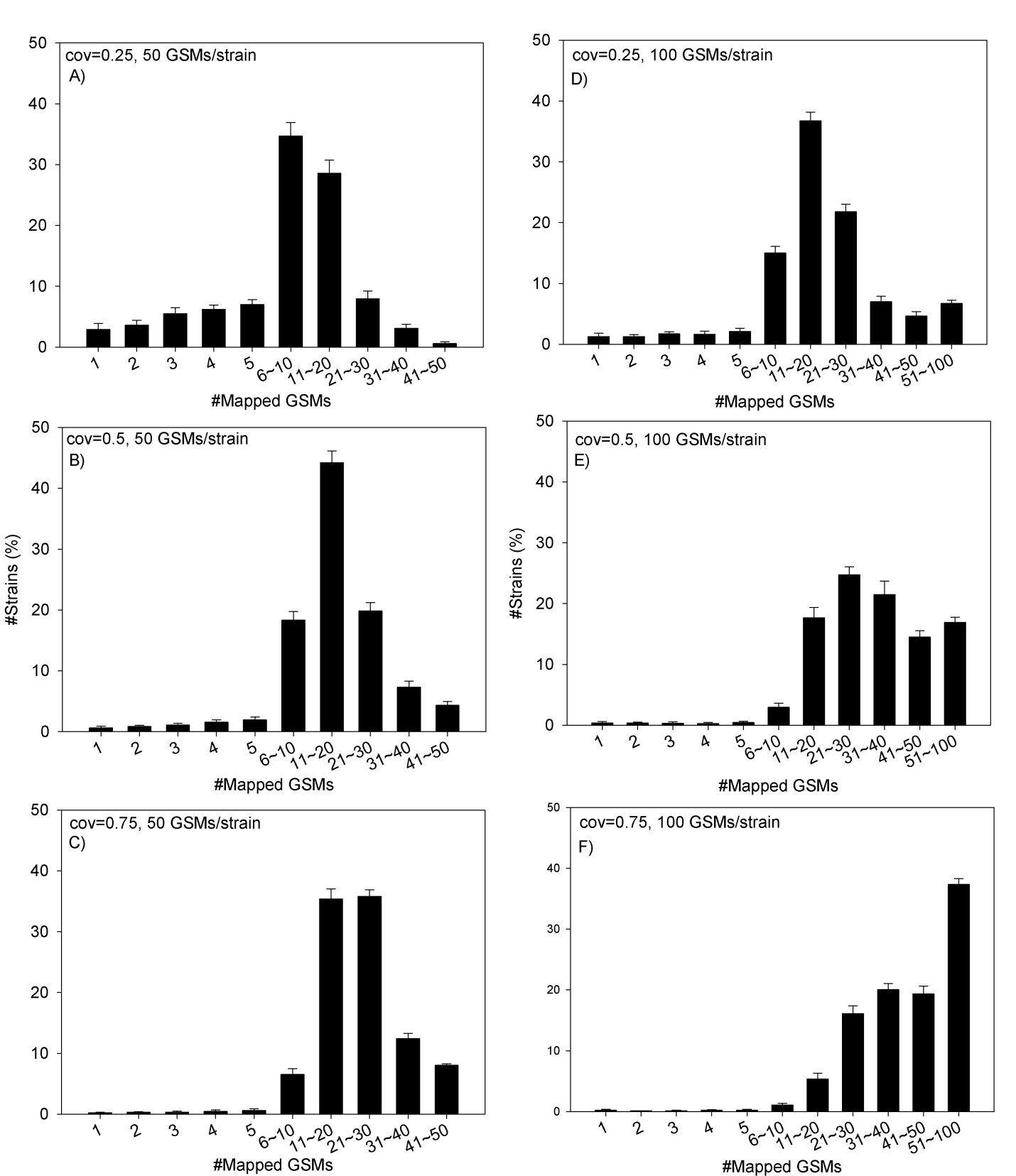


**Fig. S3** Comparison with MetaPhlAn at species level using synthetic metagenomes generated from 302 recently sequenced microbial genomes.


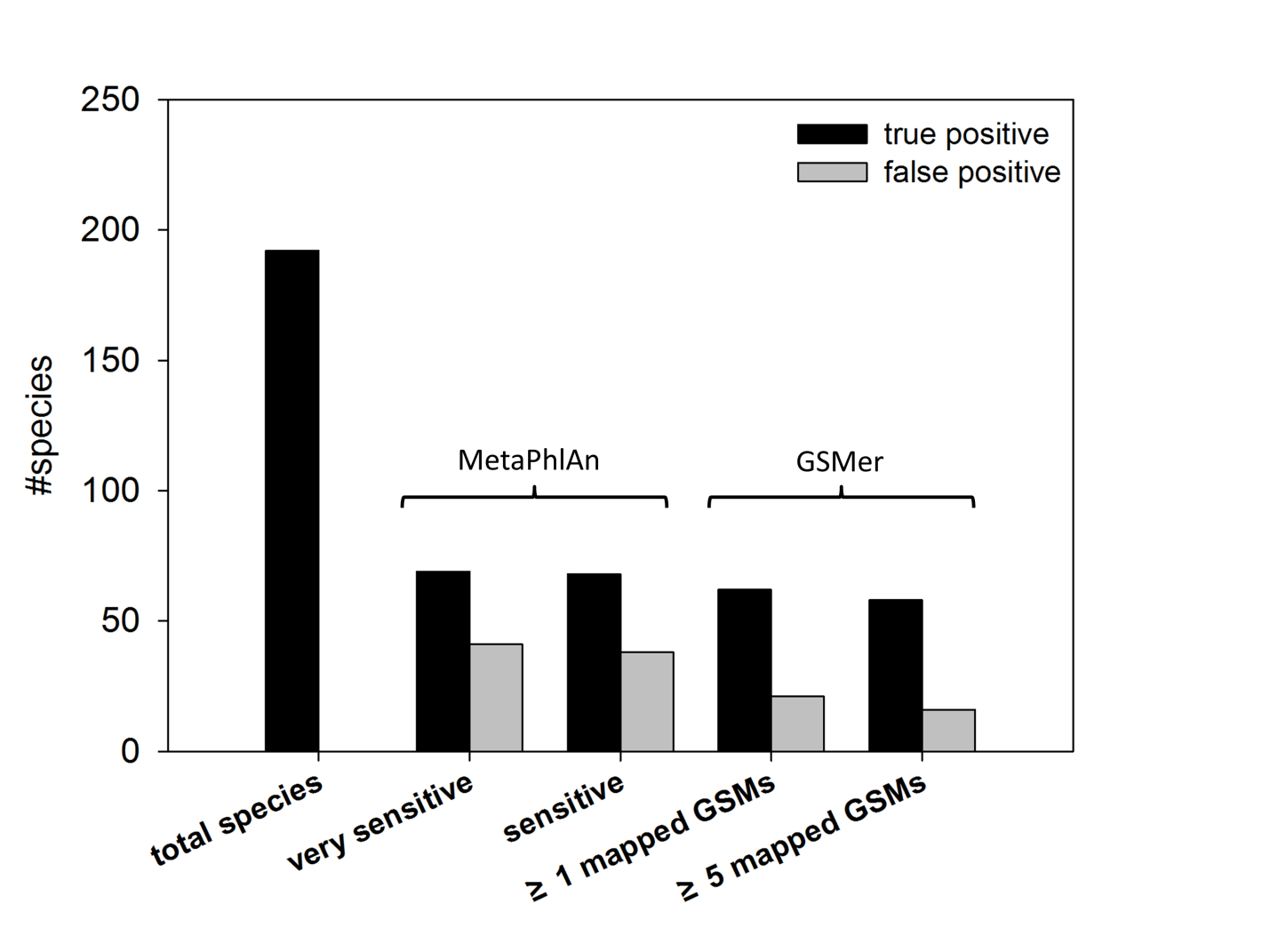

Supplement: Supplementary Data [file supp_gku138_nar-03196-met-z-2013-File006.docx]
